# Inosine: A bioactive metabolite with multimodal actions in human diseases

**DOI:** 10.3389/fphar.2022.1043970

**Published:** 2022-11-16

**Authors:** In Soo Kim, Eun-Kyoung Jo

**Affiliations:** ^1^ Department of Medical Science, Chungnam National University College of Medicine, Daejeon, South Korea; ^2^ Department of Microbiology, Chungnam National University College of Medicine, Daejeon, South Korea; ^3^ Infection Control Convergence Research Center, Chungnam National University College of Medicine, Daejeon, South Korea

**Keywords:** inosine, cancer, inflammation, infection, cardiovasclar disease, neuroprotection

## Abstract

The nucleoside inosine is an essential metabolite for purine biosynthesis and degradation; it also acts as a bioactive molecule that regulates RNA editing, metabolic enzyme activity, and signaling pathways. As a result, inosine is emerging as a highly versatile bioactive compound and second messenger of signal transduction in cells with diverse functional abilities in different pathological states. Gut microbiota remodeling is closely associated with human disease pathogenesis and responses to dietary and medical supplementation. Recent studies have revealed a critical link between inosine and gut microbiota impacting anti-tumor, anti-inflammatory, and antimicrobial responses in a context-dependent manner. In this review, we summarize the latest progress in our understanding of the mechanistic function of inosine, to unravel its immunomodulatory actions in pathological settings such as cancer, infection, inflammation, and cardiovascular and neurological diseases. We also highlight the role of gut microbiota in connection with inosine metabolism in different pathophysiological conditions. A more thorough understanding of the mechanistic roles of inosine and how it regulates disease pathologies will pave the way for future development of therapeutic and preventive modalities for various human diseases.

## Introduction

Inosine, an inert purine nucleoside, is formed by breakdown (deamination) of adenosine both intracellularly and extracellularly; it also generated by the action of 5′-nucleotidase on inosine monophosphate (IMP) (Conway and Cooke, 1939; Itoh et al., 1967; Chen et al., 1996). Recent studies have revealed that inosine is also produced by several species found in the gut microbiome and modulates host immune and inflammatory functions (Wang et al., 2020; Brown et al., 2021). Inosine can be metabolized into hypoxanthine, xanthine, and uric acid (Sorensen, 1970; Zoref-Shani et al., 1988; Doyle et al., 2018; Garcia-Gil et al., 2021). Cell membrane transport of inosine is mediated by equilibrative and concentrative nucleoside transporters (Körber et al., 1975; Belt et al., 1993; Cass et al., 1999; Miller et al., 2021). Inosine functions are mediated in receptor-dependent or–independent manners. The receptor-mediated function of inosine is thought to be related to adenosine receptor family members including A_1_, A_2A_, A_2B_, and A_3_ G-protein coupled receptors (Kypson and Hait, 1976; Fredholm et al., 2001; Fredholm et al., 2011; Welihinda et al., 2018). Compared with the known role of adenosine as a signaling molecule, the function of inosine in the context of physiological and pathological responses in human health and diseases remain poorly understood.

Earlier studies suggested that the inert purine nucleoside inosine has neuroprotective, cardioprotective, and immunomodulatory effects in different experimental models (Aviado, 1983; Haskó et al., 2004; Guinzberg et al., 2006). The beneficial function of inosine has been thought to be mediated through modulation of oxidative stress and inflammatory responses (Haskó et al., 2004). More recent studies have revealed therapeutic effects of inosine in motor function improvement during neurologic injury or stroke (Benowitz et al., 1999; Kuricova et al., 2014), learning and memory (Ruhal and Dhingra, 2018), and Parkinson’s disease (Schwarzschild et al., 2014). Inosine treatment also results in the activation of anti-tumor and anti-inflammatory responses in different disease models (Haskó et al., 2000; Mabley et al., 2003; Panebianco et al., 2018; Tobólska et al., 2018; Kovachev, 2020; Mager et al., 2020). Importantly, the beneficial effects of inosine have been demonstrated with the preclinical and clinical use of Isoprinosine (inosine pranobex), formed by inosine with the immunostimulatory dimepranol acedoben (acetamidobenzoic acid and dimethylaminoisopropanol), for treatment of neurological disorders and acute respiratory viral infections (Beran et al., 2016; Sliva et al., 2019; Teixeira et al., 2020; Beran et al., 2021; Nascimento et al., 2021; Teixeira et al., 2022a; Yang et al., 2022). In this Review, we discuss recent updates on the regulation of pathological responses by inosine and its association with gut microbiota remodeling in different contexts. Furthermore, we discuss the functions of exogenous inosine in terms of cancers, inflammation, infection, and cardiovascular and neurological diseases through its immunomodulatory roles (Table 1).

**TABLE 1:** Recent studies on inosine effects across various diseases.

Disease	Subject	Intervention	Effect	Mechanism	References
Cancers					
Cervical cancer	HPV-positive patients after cervical conization	Inosine pranobex	Reduce relapse of HSIL and high-risk HPV infection	Clear cervical HPV infection	Kovachev, (2020)
Colon cancer, bladder cancer, and melanoma	Mice with xenograft or chemical-induced tumor	Inosine	Augment immune checkpoint inhibitor efficacy	Promote Th1 immunity through activating adenosine 2A receptor	Mager et al. (2020)
Liver cancer	HepG2 cells	Inosine pranobex	Cytotoxic effect	Mitochodrial damage	Tobólska et al. (2018)
Melanoma	Mice with xenograft tumor	Inosine	Enhance immunotherapy efficacy	Support proliferation and function of effector T cells	Wang et al. (2020)
Cardiovascular diseases					
Atherosclerosis	Rats with hypercholesterolemic diet	Inosine	Alleviate atherogenic index and platelet aggregation	Activate eNOS and inhibit the NF-κB pathway	Lima et al. (2020)
Mitochondrial disease	Mt-cardiomyopathy and mt-diabetes patients	Inosine plus febuxostat	Decrease BNP and increase insulinogenic index	Enhance cellular ATP levels	Kamatani et al. (2019)
Infectious diseases					
Acute respiratory viral infection	Laboratory-confirmed viral infection patients with ILI	Insoine pranobex	Reduce time to symptom resolution	Control viral infection	Beran et al. (2016)
COVID-19	SARS-CoV-2-positive patients	Inosine pranobex	Reduce case-fatality rate	Control viral infection	Beran et al. (2020)
Influenza	Influenza A (H3N2)-infected mice	Inosine pranobex	Extend survival time with oseltamivir and ellagic acid	Protect from damaging superoxide radicals	Pavlova et al. (2018)
NTM pulmonary disease	NTM-infected mice	Inosine	Decrease bacterial loads in lungs	Enhance IFN-γ-related responses	Kim et al. (2022)
Inflammatory disease					
Acute hepatic injury	LPS-injected mice	Inosine	Suppress inflammatory cytokines and conserve liver function	Alter the microflora composition and attenuate the TLR4 pathway	Guo et al. (2021)
Alcoholic liver disease	Mice with alcohol-induced liver injury	Inosine plus LGG	Improve the liver structure and function	Suppress oxidative stress and attenuate inflammatory cytokine expression	Zhu et al. (2022)
IPEX syndrome	Scurfy mouse	Inosine	Prolong lifespan and reduce multiorgan inflammation	Inhibit Th1 and Th2 cell differentiation through adenosine A2 receptor	He et al. (2017)
NSAID-induced enteropathy	Mice with indomethacin-induced enteropathy	Inosinic acid plus pottasium oxonate	Conserve intestinal structure	Remove ROS through serum uric acid accumulation	Yasutake et al. (2017)
Sepsis	LPS-injected mice	Inosine monophosphate	Decrease TNF-α and increase IL-10	Augment inosine produced by ecto-5′-nucleotidase	Lovászi et al. (2021)
Systemic lupus erythematosus	LPS-treated human monocytes	Inosine	Inhibit autophagy and IFN-β release	Increase phosphorylated S6 and decrease phosphorylated IRF3	Wu et al. (2022)
Ulcerative colitis	Mice with DSS-induced colitis	Inosine	Protect intestinal function	Activate adenosine A2 receptor/PPAR-γ axis	Li et al. (2021b)
Neuropsychological disease					
Alzheimer’s disease	Rats with streptozotocin-induced Alzheimer’s disease	Inosine	Prevent memory deficits and weight loss	Increase BDNF and anti-inflammatory cytokines	Teixeira et al. (2022a)
Alzheimer’s disease	Rats with streptozotocin-induced Alzheimer’s disease	Inosine	Attenuate memory loss	Modulate the ion pump activities and clear the oxidative stress	Teixeira et al. (2020)
Alzheimer’s disease	Rats with scopolamine-induced cognitive impairment	Inosine	Protect from memory consolidation impairment	Modulate the ion pump and AchE activities and reduce the oxidative stress	Teixeira et al. (2022b)
Bipolar disorder	Rats with ketamine-induce mania	Inosine	Prevent hyperlocomotion behavior	Need to elucidate, not associated with adenosine receptor	Camerini et al. (2020)
CNS injury	Rat with unilateral CST transection	Contralateral inosine minipump	Stimulate axon collateral growths	Induce axon sprouting and crossing	Benowitz et al. (1999)
CNS injury	Rat with spinal cord compression	Inosine	Improve recovery of motor and urinary function	Increase axonal ramification	Kuricova et al. (2014)
Cognitive dysfunction	Aged female rats	Inosine	Elevate learning and memory function	Conserve hippocampal CA1 region with anti-inflammatory and antioxidant effect	Ruhal and Dhingra, (2018)
Diabetic peripheral neuropathy	Rats with streptozotocin and nicotinamide induced diabetes	Inosine	Recover the structure and function of the sciatic nerve	Reduce blood glucose level and oxidative stress	Abdelkader et al. (2022)
Huntington’s disease	Rats with 3-NP-induced neurotoxicity	Inosine	Mitigate the disease symptoms	Activate adenosine A2 receptor/BDNF/ERK axis	El-Shamarka et al. (2022)
Methamphetamine withdrawal syndrome	Methamphetamine-treated mice	Inosine	Restore the anxiety and depression-like behavior	Potential neuroprotective function	Yang et al. (2022)
Multiple system atrophy	Multiple system atrophy patients	Inosine monophosphate	Improve cognitive function	Increase serum uric acid	Jung Lee et al. (2021)
Parkinson’s disease	Early Parkinson’s disease patients	Inosine	Mitigate the disease progression	Increase cerebrospinal fluid urate	Schwarzschild et al. (2014)
Parkinson’s disease	Parkinson’s disease patients	Inosine plus febuxostat	Improve disease symptoms	Increase blood hypoxanthine and xanthine but decrease uric acid	Watanabe et al. (2020)
Parkinson’s disease	Early Parkinson’s disease patients	Inosine	No significant difference in the disease progression	—	Schwarzschild et al. (2021)
PNS injury	Mice with sciatic nerve crush	Inosine	Accelerate axonal regeneration and functional recovery	Reduce the number of macrophages and myelin ovoids	Soares Dos Santos Cardoso et al. (2019)

**Abbreviation**: 3-NP, 3-nitropropionic acid; AChE, acetylcholinesterase; ATP, adenosine triphosphate; BDNF, brain-derived neurotrophic factor; BNP, brain natriuretic peptide; CNS, central nervous system; CST, corticospinal tract; DSS, dextran sulfate sodium; eNOS, endothelial nitric oxide synthase; ERK, extracellular signal-regulated kinase; HSIL, high-grade squamous intraepithelial lesion; HPV, human papilloma virus; IFN, interferon; IL, interleukin; ILI, influenza-like illnesses; IPEX syndrome, immune dysregulation, polyendocrinopathy, and enteropathy, with X-linked inheritance; IRF3, interferon regulatory factor 3; LGG, *Lactobacillus rhamnosus* GG; LPS, lipopolysaccharide; Mt, mitochondria; NF-κB, nuclear factor-κB; NSAID, nonsteroidal anti-inflammatory drug; NTM, nontuberculous mycobacteria; PNS, peripheral nervous system; PPAR, peroxisome proliferator-acitvated receptor; ROS, reactive oxygen species; TLR4, toll-like receptor 4; TNF, tumor necrosis factor.

## Overview of inosine biology

**FIGURE 1:**
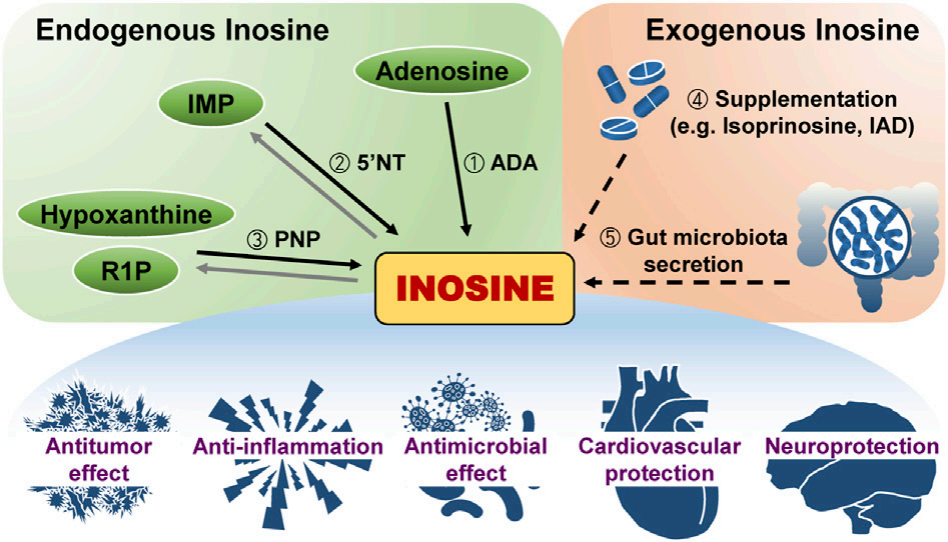
Inosine generation in the body and its effect. Inosine originates either within or outside the body. Three different reactions produce endogenous inosine: ①Hydrolytic deamination of adenosine by adenosine deaminase (ADA), ② dephosphorylation of inosine monophosphate (IMP) by 5′-nucleotidase (5′NT), and ③ reaction of hypoxanthine and ribose-1-phosphate (R1P) by purine nucleoside phosphorylase (PNP). Exogenous inosine is from two ways: ④ Supplementation such as isoprinosine or inosine acedoben dimepranol (IAD) or ⑤ secretion from gut microbiota. Inosine has various effects on the body.

Inosine, an intermediate in purine metabolism, consists of hypoxanthine and ribose. Three enzymatic reactions can form inosine endogenously (Figure 1). First, adenosine deaminase (ADA) irreversibly removes the amine group of adenine ring intracellularly and extracellularly (Akedo et al., 1972; Van Heukelom et al., 1976; Van der Weyden and Kelley, 1977; Akeson et al., 1988). In particular, double-stranded RNA (dsRNA)-specific adenosine deaminases (ADARs) mediate adenosine-to-inosine editing (Liddicoat et al., 2015; George et al., 2016; Eisenberg and Levanon, 2018; Nakahama and Kawahara, 2020; Pfaller et al., 2021). ADAR1 and ADAR2 maintain balanced immune activation and self-tolerance through inhibiting dsRNA-binding proteins, such as RIG-I-like receptors, protein kinase R, and oligoadenylate synthases-RNAse L (Yang et al., 2014; Liddicoat et al., 2015; George et al., 2016; Jain et al., 2019; Lamers et al., 2019; Schaffer et al., 2020; Pfaller et al., 2021; Li et al., 2022). In a recent study, the severe autoinflammatory disease Aicardi–Goutières syndrome mouse model with *ADAR1* mutation reveals immunopathology associated with type I interferon signaling and Z-binding protein 1 (Rice et al., 2012; de Reuver et al., 2022). Contrary to ADAR1 and ADAR2, ADAR3 suppresses A-to-I editing by binding to RNA-binding domains (Chen et al., 2000; Oakes et al., 2017; Raghava Kurup et al., 2022). Second, 5′-nucleotidase catalyzes the reversible dephosphorylation of IMP both inside and outside cells (Barsotti et al., 2005; Ipata and Balestri, 2013). Cytosolic 5′-nucleotidase (NT5C2) is correlated with chemotherapy resistance in acute lymphoblastic leukemia (Tzoneva et al., 2013). Ecto-5′-nucleotidase (NT5E, CD73) mediates immune suppression (Romio et al., 2011; Lovászi et al., 2021). Third, purine nucleoside phosphorylase (PNP) converts hypoxanthine and ribose-1-phosphate (R-1-P) into inosine and thermodynamically favors this enzymatic synthesis over phosphorolysis (Kalckar, 1947; Friedmin, 1950; Tozzi et al., 2006). However, the PNP reaction equilibrium is biased toward inosine degradation due to the more significant concentration of inorganic phosphate than base and R-1-P (Traut, 1994), and the linked reaction of hypoxanthine catalyzed by hypoxanthine phosphoribosyl transferase and xanthine oxidase (Pugmire and Ealick, 2002; Il’icheva et al., 2020). Xanthine oxidase catabolizes hypoxanthine into uric acid *via* xanthine (Heinz et al., 1980; Moriwaki et al., 1999; Doyle et al., 2018). Humans and higher primates excrete uric acid in their urine, but other mammals convert uric acid to allantoin by uricase and then excrete it in urine (Heinz et al., 1980; Kurtz et al., 1986; Moriwaki et al., 1999). Uric acid, the end product of human purine metabolism, is one of the major antioxidants (Ames et al., 1981; Whiteman et al., 2002; Muraoka and Miura, 2003) and protects against neurological and intestinal diseases (Hooper et al., 1998; Hooper et al., 2000; Toncev et al., 2002; Ascherio et al., 2009; Matsuo et al., 2015; Yasutake et al., 2017). Consequently, inosine-related metabolism should be extensively explored for its multidimensional effects on human illnesses.

In the human gut, 10^13^–10^14^ microorganisms are found and are emerging as important players in the pathogenesis and therapeutics of a variety of human diseases (Wardman et al., 2022). Recently, a mechanistic link between microbiota and inosine has been revealed. For example, the microbiome-derived Bxa, an abundant ADP-ribosyltransferase (ADPRT) of *Bacteroides*, induced secretion of inosine as a carbon source, thus acting as a bacterial fitness factor (Brown et al., 2021). Gut microbiota remodeling enriched *Bifidobacterium pseudolongum* and supplementation with its metabolite inosine increased the anti-tumor effects of immune checkpoint blockade and functioned as a carbon source for CD8^+^ effector T-cell function (Wang et al., 2020). In a more recent study, inosine was found to be a microbiota-derived immunostimulatory metabolite that enhanced immunotherapeutic effects and antitumor T-cell responses (Kroemer and Zitvogel, 2020; Mager et al., 2020). Indeed, gut microbiota remodeling enriched *B. pseudolongum* significantly increased the anticancer immunotherapy response through the generation of inosine *via* A_2A_ receptors (Mager et al., 2020).

## A multifaceted role for inosine in cancer

Multiple studies have suggested that inosine functions as a crucial biomarker metabolite associated with cancer metastasis, drug resistance and/or treatment, and tumor progression (Li et al., 2021a; Lee et al., 2021; Stockard et al., 2021). A recent study showed that inosine can predict the metastatic potential of lung squamous cell carcinoma (Lee et al., 2021). Inosine has also been shown to be associated with acute myeloid leukemia with cytarabine resistance (Stockard et al., 2021). In esophageal squamous cell carcinoma, inosine has been associated with cancer progression (Li et al., 2021a). In colorectal cancer organoids, inosine was elevated following treatment with 5-fluorouracil (Neef et al., 2020), although the underlying mechanism is unclear. In head and neck squamous cell carcinoma, inosine and adenosine are abundant purine metabolites present in exosomes from the supernatant of UMSCC47 cells (Ludwig et al., 2020). Inosine is a crucial serum metabolite that can differentiate between low- and high-grade bladder cancer patients (Tan et al., 2017). Moreover, urine and serum levels of inosine as well as other metabolites can be used to characterize hepatocellular carcinoma patients (Chen et al., 2011; Ladep et al., 2014). These data suggest that inosine has a promising role in the diagnosis and grading of tumors as a signature metabolite. By contrast, inosine levels are generally decreased in pancreatic cancer patients, and elevated by a low carbohydrate ketogenic diet, compared to a general hospital diet (Kang et al., 2019). These data collectively suggest that inosine functions as a potential biomarker for prediction of cancer risk, drug response, and early detection of metastasis of various tumors.

Several recent studies have indicated the role of inosine in anti-tumor responses in various cancers. In a xenograft model of pancreatic ductal adenocarcinoma, gemcitabine-mediated chemotherapy significantly induced activation of nuclear factor (NF)-κB-mediated inflammatory responses and decreased the proportion of *Firmicutes* and *Bacteroides*. The chemotherapy also suppressed serum levels of inosine and xanthine (Panebianco et al., 2018), suggesting chemotherapy may influence gut microbiota remodeling and changes in serum metabolites. Moreover, inosine supplementation promoted immune checkpoint blockade and provided a carbon source for CD8^+^ effector T-cell function (Wang et al., 2020). In a more recent study, inosine was found to be a microbiota-derived immunostimulatory metabolite that enhanced immunotherapeutic effects and antitumor T-cell responses (Kroemer and Zitvogel, 2020; Mager et al., 2020). Indeed, gut microbiota remodeling enriched *B. pseudolongum* and significantly increased the effects of immunotherapy through the generation of inosine *via* A_2A_ receptors (Mager et al., 2020).

Although inosine pranobex (isoprinosin) has been shown to exhibit significant antiviral effects (Beran et al., 2021), few studies have investigated its effects in cancer. Treatment with isoprinosin significantly increased the clearance of cervical human papillomavirus (HPV) infection during postoperative immunotherapy in women receiving surgical treatment for high-grade squamous intraepithelial cervical lesions (Kovachev, 2020). Addtionally, a previous study revealed increased cytotoxicity of fibroblasts and hepatocellular carcinoma HepG2 cells following inosine pranobex treatment (Tobólska et al., 2018). Because inosine pranobex is useful in viral infections and has remarkable immunomodulating functions in both cellular and humoral immune responses (Kovachev, 2021), the therapeutic application of inosine pranobex is challenging in terms of virus-related tumors accompanied with chronic inflammation and immunosuppression. Although it would have been shown to be therapeutically beneficial in animal studies, the use of inosine pranobex in anticancer regimens should be considered following more data from clinical trials.

## Inosine and antimicrobial immunity

In nonhuman primate models infected with human immunodeficiency virus type 1/simian immunodeficiency virus (HIV-1/SIV), elevated inosine levels have been related to immune activation and disease progression markers (He et al., 2015). The disease-progressive model reveals higher ADA activity and CD26 expression on intestinal T cells than the nonprogressive model, suggesting that adenosine degradation stimulates T cells (He et al., 2015). In addition, several recent studies have suggested a link between inosine and antimicrobial function during bacterial infection. Our recent study showed that inosine, as a metabolite of *B. pseudolongum*, contributes to antimicrobial responses in mouse models with *Mycobacteroides abscessus* (Mabc) infection (Kim et al., 2022). Interestingly, ʟ-arginine administration of Mabc-infected mice led to gut microbiota remodeling toward enrichment of *B. pseudolongum*, which promoted effector T-cell responses with IFN-γ activation and inducible nitric oxide synthase (iNOS) expression, indicating a Th1-mediated M1 shift (Kim et al., 2022). Importantly, ʟ-arginine administration upregulated the serum level of inosine in mice infected with Mabc, and inosine treatment exhibited a similar protective phenotype as observed in ʟ-arginine-treated conditions in the context of NTM infections (Sun et al., 2020; Kim et al., 2022). It was previously reported suberic acid is produced by *B. pseudolongum* (Sun et al., 2020); however, treatment of Mabc-infected mice with suberic acid did not show any protective immune responses during Mabc infection. These data strongly suggest that inosine, produced by *B. pseudolongum*, plays a distinct role in the enhancement of antimicrobial responses during NTM infection.

Isoprinosine appears to be an effective treatment for various viral infections through pleiotropic immunomodulatory roles including T-cell activation and proinflammatory cytokine-mediated functions (Sliva et al., 2019; Beran et al., 2021). Isoprinosine combined with antiviral and antioxidant drugs has been shown to produce a protective effect, with increased survival and decreased lung pathologies, in influenza H3N2 virus-infected mice (Pavlova et al., 2018). In addition, a Phase 4 clinical study showed that isoprinosine is safe for treatment of patients with acute respiratory viral infections and is effective in the resolution of influenza-like symptoms in individuals less than 50 years of age (Beran et al., 2016). A preliminary report emphasized the effectiveness of inosine pranobex in the reduction of the case-fatality rate in older COVID-19 patients in the Czech Republic (Beran et al., 2020), although larger-scale trials are warranted to clarify the therapeutic effects of inosine pranobex against COVID-19. Inosine acedoben dimepranol (IAD), another licensed inosine-based drug, has been shown to boost NK cell numbers in clinical trials (Rumel Ahmed et al., 2017). Future clinical studies are needed to determine whether isoprinosine or IAD can be effectively used as adjunctive drugs in antiviral therapeutics without overt complications.

## Inosine and therapeutic implications for inflammation

A recent report showed that inosine, metabolized from IMP, suppressed tumor necrosis factor (TNF)-α *in vitro* and *in vivo*, although A_2A_, A_2B_, and A_3_ receptors were not involved (Lovászi et al., 2021). In addition, inosine augmented IL-β production in response to NLRP3 inflammasome stimuli in macrophages (Lovászi et al., 2021). These data suggest that inosine is involved in the activation or suppression of inflammatory responses, depending on the stimuli.

Several studies have shown the combined beneficial effects of inosine and probiotics in experimental inflammatory disease models. For example, *Lactobacillus rhamnosus* GG (LGG) combined with inosine has been shown to ameliorate hepatic inflammation and restore regulatory T-cell function in an alcohol-induced liver injury model (Zhu et al., 2022). Importantly, combined treatment of LGG and inosine significantly improved immune homeostasis and intestinal microecology with amelioration of gut dysbiosis during liver injury (Zhu et al., 2022). Interestingly, inosine treatment can alter gut microbiota toward an abundance of *Bifidobacterium* and *Lachnospiraceae* UCG-006 and suppress TLR4 signaling, thus negatively regulating hepatic inflammation and damage (Guo et al., 2021).

In the context of colitis, dietary barley leaf supplementation has been shown to produce anti-inflammatory effects in dextran sulfate sodium (DSS)-induced colitis and dysbiosis of the gut microbiome. Mechanistically, dietary barley leaf elicits inosine accumulation in colonic epithelial cells through the alteration of gut bacterial composition, such as an abundance of *Lactobacillus* (Li et al., 2021b). In addition, the exogenous inosine administration showed a similar protective effect on colitis through the activation of A_2A_ receptor and peroxisome proliferator-activated receptor (PPAR)-γ signaling (Li et al., 2021b). Furthermore, indomethacin-induced enteropathy is ameliorated by inosinic acid (1,000 mg/kg, i.p.) in mice. Inosinic acid treatment leads to increased serum levels of uric acid, resulting in protection against intestinal injury *via* antioxidative effects (Yasutake et al., 2017).

A recent study showed that NAD + precursor nicotinamide riboside (NR) suppresses lipopolysaccharide-induced IFN-β production and autophagy activation in myeloid cells at least partly through inosine-mediated signaling (Wu et al., 2022). Importantly, NR indicated anti-inflammatory effects in monocytes from patients with the autoimmune disorder systemic lupus erythematosus (SLE), showing dysregulated type I IFN production (Wu et al., 2022). Plasma inosine increase *via Limosilactobacillus reuteri* (LR) administration exhibits a protective effect in the scurfy mouse, lacking regulatory T cells, that mimics human IPEX syndrome (immune dysregulation, polyendocrinopathy, and enteropathy, with X-linked inheritance) (He et al., 2017; Liu et al., 2021). LR reverses decreased inosine levels in plasma from scurfy mice and impacts the amelioration of disease pathologies and multiorgan inflammation through A_2A_ receptors (He et al., 2017; Liu et al., 2021). Interestingly, the ability of LR to increase plasma inosine is unique compared to the probiotic LGG (Liu et al., 2021). These data suggest inosine may act as a bioactive molecule responsible for distinct anti-inflammatory properties in immunodeficiency and autoimmune diseases.

## Inosine and other diseases

### Inosine and cardiovascular diseases

Inosine is thought to be as “a coronary dilator” through relaxation of the coronary artery and inotropic action, although the mechanisms of action remain unclear (Aviado, 1983). Alternatively, the role of ADA competitive inhibition by inosine cannot be excluded. In addition, accumulating evidence suggests that adenosine has a protective role against endothelial dysfunction and vascular inflammation (Kutryb-Zajac et al., 2020). Dysregulated ADA activity leads to cardiovascular pathologies such as atherosclerosis, thrombosis, and myocardial ischemia-reperfusion injury (Kutryb-Zajac et al., 2020), suggesting that ADA may be a critical target for treatment of cardiovascular disease. Interestingly, co-administration of febuxostat and inosine exhibited a favorable response in two patients with mitochondrial diseases, cardiomyopathy and diabetes (Kamatani et al., 2019).

In a hypercholesterolemic rat model, inosine treatment improved endothelium-mediated vasodilatation and antiplatelet function (Lima et al., 2020). A recent study suggested that urinary inosine is inversely associated with coronary heart disease risk in men (Yoon et al., 2020). Several metabolites including inosine are prominent biomarkers in Kawasaki disease patients with coronary artery lesions (Qian et al., 2021). The existence of brown adipose tissue (BAT) is an independent factor for lowering cardiovascular and metabolic disease (Becher et al., 2021). Extracellular inosine enhances the energy expenditure of BAT in mice. In addition, loss of function mutation of human *SLC29A1*, which decreases extracellular inosine levels, is strongly correlated with lean body mass and non-obese (Niemann et al., 2022). These data underpin the protective effects of inosine on the cardiovascular system.

### Inosine in psychiatric and neurological pathologies

Recent studies have shown neuroprotective and neuromodulatory functions of inosine in a variety of neurological and psychiatric diseases (Nascimento et al., 2021). Inosine treatment ameliorated 3-nitropropionic acid (3-NP)-induced neurotoxicity and boosted brain-derived neurotrophic factor (BDNF) levels, p-cAMP response element-binding protein (CREB) expression, and glutathione content (El-Shamarka et al., 2022). In addition, metformin-mediated amelioration of methamphetamine withdrawal syndrome is at least partly mediated through altered bacterial composition and metabolite changes such as abundance of *Rikenellaceae* and inosine (Yang et al., 2022). Interestingly, inosine supplementation of mice improved methamphetamine withdrawal-mediated anxiety and depression-like symptoms (Yang et al., 2022), indicating that metformin effects may depend on microbiota-derived inosine.

In rat models of Alzheimer’s disease, inosine treatment has been shown to prevent memory deficits, suppress immunoreactivity *via* brain A_2A_ receptor, and enhance anti-inflammatory cytokine levels and oxidative alterations in the brain (Teixeira et al., 2020; Teixeira et al., 2022a). In addition, inosine administration had beneficial effects in a rat model of diabetic peripheral neuropathy, resulting in a hypoglycemic effect and enhanced myelination (Abdelkader et al., 2022). Inosine-induced neuroprotective function depends on Nrf2 expression, downstream HO-1, and suppression of PKC and TRPV1 (Abdelkader et al., 2022). Inosine treatment also resulted in elevated memory acquisition and consolidation in a rat model of scopolamine-induced cognitive impairment. These effects are partly mediated through modulation of brain redox status, cholinesterase function, and ion pump activity, suggesting promising approaches for neurodegenerative diseases (Teixeira et al., 2022b). In a ketamine-induced rat mania model, inosine administration may prevent hyperlocomotion and attenuation of maniac phase symptoms (Camerini et al., 2020). In an experimental model of sciatic nerve crush injury in mice, inosine treatment resulted in accelerated axonal regeneration and a recovery of motor and sensory functions (Soares Dos Santos Cardoso et al., 2019).

A clinical trial with co-administration of febuxostat and inosine showed improvement of the symptoms of Parkinson’s disease (Watanabe et al., 2020). However, another recent clinical trial showed that inosine treatment does not modify progression of early Parkinson’s disease (Schwarzschild et al., 2021). In a clinical trial for use of IMP in multiple system atrophy (MSA), IMP raised serum levels of uric acids, and was considered to be tolerable and safe during a 24-week treatment (Jung Lee et al., 2021), although a more long-term follow-up study is warranted.

## Concluding remarks

Recent advances have contributed to our understanding of the role of inosine as a biomarker or a regulator of immunity, infection, inflammation, cancer, and other pathological conditions. In addition, accumulating reports suggest that the levels of inosine or inosine-related enzymes are dysregulated in diseases such as cancers. More studies are needed to identify how inosine levels are regulated in different tissues under various physiological and pathological conditions. The information available on the impact of inosine on immunomodulatory and protective functions has identified this metabolite as a possible therapeutic target for tumors, inflammation, infection, and cardiovascular and neurological disorders. Future studies with preclinical models and clinical research are required to bring the inosine-based therapeutic approach into the clinic.

Several microbes from the intestinal microbiota have been reported to produce inosine, exhibiting therapeutic efficacy in various disease models. Future molecular studies will be important to reveal how the link between the microbiota and inosine regulates immune and inflammatory responses in specific human diseases.

The use of isoprinosine, a synthetic agent of inosine with an immunostimulant, in several clinical trials has suggested that inosine or inosine-based drugs may be therapeutically useful in neurological and autoimmune diseases. However, most data regarding the physiological and therapeutic potential of inosine have been derived from experimental studies. There are also concerns for the possibility of uric acid stones, which can be produced by inosine metabolism. Therefore, another strategy based on the regulation of inosine-degrading enzymes (i.e., PNP) could be also useful for potential treatment targeting inosine-based therapeutics. Future preclinical and clinical trials are needed to assess the safety and adverse effects associated with long-term use of inosine or inosine-related enzyme modulation.

To date, there is limited information on the molecular mechanisms through which inosine exerts its biological functions as a signaling mediator. Evidence suggests that inosine may function through multiple subtypes of adenosine receptors. However, it remains unclear how inosine targets adenosine receptor subtypes or whether there are alternative inosine receptors in different cell types. Understanding the pathways responsible for the dysregulation of inosine production and finding inosine-specific effectors will facilitate the development of therapeutic strategies against various disorders.
